# SATB1 Exhibits a Protective Role in HAmylin‐Oligomer‐Induced Neuronal Damage and Cognition Decline

**DOI:** 10.1002/cns.70781

**Published:** 2026-02-13

**Authors:** Yuan Xing, Wei Zhang, Zuxiao Yang, Xia Qin, Ting Zhou, Nan Zhang

**Affiliations:** ^1^ Neuromedical Technology Innovation Center of Hebei Province, Department of Neurology The First Hospital of Hebei Medical University Shijiazhuang Hebei China; ^2^ Department of Pharmacology, Institution of Chinese Integrative Medicine Hebei Medical University Shijiazhuang Hebei China; ^3^ Department of Neurology, Xinqiao Hospital The Army Medical University (Third Military Medical University) Chongqing China

**Keywords:** AKT3, cognitive ability, hAmylin oligomers, neuronal apoptosis, SATB1

## Abstract

**Aims:**

Amylin is a hormone secreted by pancreatic β cells, and oligomerized human amylin (hAmylin) has shown neurotoxicity in mice. However, the effect of hAmylin oligomers on cognitive ability and the underlying mechanisms remains unexplored.

**Methods:**

In this study, hAmylin oligomers were bilaterally injected into the hippocampus of mice.

**Results:**

Inspiringly, hAmylin‐mice displayed obvious cognition decline and neuronal damages in the hippocampal dentate gyrus (DG) area. Thus, hippocampal tissues were collected for mRNA‐seq analysis, which identified the upregulation of transcription factor SATB Homeobox 1 (SATB1) in the hippocampus of hAmylin mice. Using behavioral experiments and histopathological analyses, the promoting effect of SATB1 knockdown on cognition decline and neuronal damage in hAmylin mice was determined. In vitro, SATB1 expression was reduced in primary hippocampus neurons after hAmylin oligomer incubation. SATB1 overexpression inhibited hAmylin‐induced mitochondrial dysfunction and neuronal apoptotic deaths, while SATB1 knockdown exhibited opposite effects. SATB1 transcriptionally activated AKT serine/threonine kinase 3 (AKT3) by binding to its promoter region, and AKT3 accounted for SATB1‐mediated protection against hAmylin‐induced mitochondrial dysfunction and neuronal injury.

**Conclusion:**

In summary, this study highlights the novel role of SATB1 in hAmylin oligomer‐induced neuronal damage and cognition decline via AKT3.

## Graphical abstract

In this study, bilateral hippocampal injections of hAmylin oligomers induced an obvious cognition decline in mice, along with upregulation of SATB1 in the mouse hippocampus. In hAmylin‐induced mice, SATB1 knockdown (KD) further aggravated the above changes. In hAmylin‐induced neurons, SATB1 KD promoted hAmylin–induced mitochondrial dysfunction and apoptosis, while SATB1 overexpression exhibited the opposite effect. Besides, SATB1 transcriptionally activated AKT3 to protect against these hAmylin‐induced disorders.

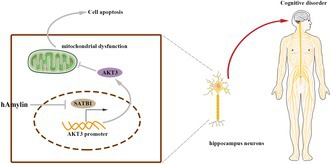

## 1 Introduction

Amylin was first discovered in 1986 and acknowledged as a hormone composed of 37 amino acids [1]. It is secreted by pancreatic beta cells together with insulin [2] and has shown the property to mediate peripheral energy homeostasis [3] [4] by inhibiting glycogen synthesis and glucose uptake [5, 6]. In addition to the peripheral effects, amylin could penetrate the blood–brain barrier, thereby participating in the regulation of the central nervous system [7, 8]. It is worth noting that the deposition of amylin has been observed in the brain of patients with type 2 diabetes and Alzheimer's disease (AD) [4]. Human Amylin (hAmylin) accumulation results in obvious neural degeneration in the rat hippocampus following a high‐fat diet [9]. Rats overexpressing hAmylin not only exhibit elevated blood glucose, but also exhibit neuroinflammation and cognitive dysfunction [10]. hAmylin exacerbates the deposition of Aβ_42_ in the brain, leading to cognitive impairment and synapse reduction [11]. More importantly, the oligomerization of amylin is believed to be the major cause of its neurotoxicity [10], as evidenced by that intraventricular injections of hAmylin oligomers induce significant neuron loss in the hippocampus [12]. However, the effect of hAmylin oligomers on cognitive ability has not been explored yet.

In this study, hAmylin oligomers were bilaterally injected into the hippocampus of mice, and transcription factor SATB Homeobox 1 (SATB1) was identified to be upregulated in the mouse hippocampus 4 weeks after hAmylin injection. SATB1 is a crucial factor that maintains brain functions [13, 14]. SATB1 alleviates neuronal ferroptosis in cerebral ischemic mice [15] and drives cell senescence in dopaminergic neurons after its functional loss [16, 17]. Besides, SATB1 is involved in the regulation of Ca^2+^‐signals [18] and mitochondrial reactive oxygen species (ROS) production [19], two contributors to apoptosis‐related signaling. However, the role of SATB1 in hAmylin oligomer‐induced neuronal damage and cognition decline is yet to be fully explored. As a member of the SATB Homeobox transcription factor family, SATB1 has been reported to transcriptionally activate gene expression by binding to specific motifs. In detail, SATB1 activates HuD transcription by binding to its promoter region, therefore promoting neuronal differentiation [20]. SATB1 advances CCL2 expression to induce macrophage migration via binding to its DNA [21]. In this study, AKT serine/threonine kinase 3 (AKT3) was identified as a potential target of SATB1 in hAmylin oligomer‐induced neurons. It is reported that AKT3 knockout mice manifest impaired spatial cognition [22], while AKT3 overexpression displays a neuroprotective role in familial amyotrophic lateral sclerosis [23]. Additionally, AKT3 is crucial for preserving normal mitochondrial function [24]. We thus speculate that SATB1 may alleviate hAmylin‐induced cognition decline and neuronal damage via transcriptionally activating AKT3.

## 2 Materials and Methods

### 2.1 Animal Studies

Due to the effect of estrogen changes on cognitive performance [25, 26], only male C57BL/6 mice (aged 3 months [12]) were used in this study to enhance the reproducibility of experimental results and avoid the fluctuations caused by hormone changes. In detail, mice were injected with 120 pmol/μl of hAmylin into the bilateral hippocampus. The injection (5 μL for each side) was performed at a rate of 1 μL/min in reference to the reported literature [27, 28]. Mice in the control group were injected with the same volume of normal saline. Four weeks later, the cognitive ability of mice was evaluated through the Y maze and Morris water maze test. To evaluate the effect of SATB1 on cognitive performance in hAmylin‐induced mice, recombinant adeno‐associated virus serotype 9 (AAV9) expressing enhanced green fluorescent protein (eGFP) and corresponding shRNA were generated (AAV9‐shSATB1‐eGFP and AAV9‐shNC‐eGFP). Two weeks before hAmylin oligomer injection, the mice received bilateral hippocampal injections (BHI) of AAV9 virus (1 × 10^11^ vg in 2 μL) at a rate of 0.2 μL/min. Four weeks after hAmylin injection, the mice underwent the above behavioral experiments. All animal experiments were performed with the approval of the Ethics Committee of the First Hospital of Hebei Medical University (S00668).

### 2.2 Behavioral Experiments

#### 2.2.1 Y Maze Test

To evaluate short‐term working memory, the mice were placed individually at the distal end of one arm in the Y maze, facing the intersection. They were allowed to explore the Y maze for 8 min, and the total number of entries into each arm was recorded for each mouse. The percentage of alteration was calculated using the following formula: Alteration % = (Number of alterations)/ (Total number of arm entries‐2) × 100% [29]. To evaluate spatial reference memory, the mice were placed individually into one arm (starting arm), facing the intersection. They were allowed to explore the maze for 15 min with one arm closed (new arm). One hour later, the blocked arm was opened, and the same mice were placed into the distal part of the starting arm, allowing the mice to explore the maze for 5 min.

#### 2.2.2 Morris Water Maze Test

On the first day of the experiment, a visible platform was used to screen out qualified mice. From the second day to the sixth day, the mice were subjected to navigation training with a hidden platform. The escape latency and swimming distance were recorded. On the seventh day, the mice were assigned to the spatial exploration test, with the platform removed. The number of platform crossings and target quadrant residence time within 60 s were recorded.

### 2.3 TUNEL and Immunofluorescence (IF) Staining

Hippocampus tissue was paraffin‐embedded and cut into 5‐μm sections. Next, the section was deparaffinized in xylene and rehydrated in ethanol. For TUNEL staining, the section was permeabilized with 0.1% Triton X‐100 (diluted in citric acid‐sodium citrate buffer solution) for 8 min at room temperature and incubated with TUNEL reaction solution (Roche, Switzerland) for 60 min at room temperature away from light.

4′,6‐diamidino‐2‐phenylindole (DAPI) counterstaining was next performed for 5 min in the dark. Representative TUNEL‐stained images were captured under a microscope. For NeuN/SATB1 IF staining, the deparaffinized section was immersed in antigen retrieval solution, blocked with 1% bovine serum albumin (BSA), and incubated with anti‐NeuN antibody (26975‐1‐AP, Proteintech, Wuhan, China) and anti‐SATB1 antibody (PA5‐20535, Invitrogen, USA) at 4°C overnight. The next day, secondary antibody incubation was conducted at room temperature for 1 h. DAPI was used for nuclear counterstaining, and representative photographs were taken under a microscope. To measure cell apoptosis in vitro, cells were permeabilized with 0.1% Triton X‐100, incubated with TUNEL reaction solution, and counterstained with DAPI. For cellular SATB1 detection, the cells that underwent permeabilization were blocked with 1% BSA, incubated with anti‐SATB1 antibody (PA5‐20535, Invitrogen, Wuhan, China), and immersed in secondary antibody. Representative IF images were obtained after DAPI counterstaining.

### 2.4 Nissl Staining

Hippocampal deparaffinized sections were stained with 0.5% cresol violet solution for 15 min and immersed in 0.25% ethanol–acetic acid solution (250 μL of glacial acetic acid was dissolved in 100 mL 95% ethanol) for a few seconds. After dehydration and transparency, the section was sealed and subjected to photographic microscope analysis.

### 2.5 Fluoro‐Jade B Staining

Hippocampal deparaffinized sections were immersed in 80% ethanol containing 1% NaOH for 5 min and 70% ethanol for 2 min. Subsequently, the section was incubated with 0.06% potassium permanganate for 10 min on a shaker. Following washing with distilled water for 2 min, the section was stained with Fluoro‐Jade B solution (AG310‐30MG, Merck Millipore) for 20 min at room temperature, shielding from light. After drying, the sealed section was observed under a microscope.

### 2.6 Immunohistochemistry

Hippocampal sections were incubated with antigen retrieval solution for 10 min and treated with 3% H_2_O_2_ for 15 min. After blocking with 1% BSA, the section was incubated with SATB1 (PA5‐20535, Invitrogen, USA) antibody at 4°C overnight and goat anti‐rabbit IgG‐HRP (D110058, Sangon, China) for 1 h at room temperature. 3′3′‐Diaminobenzidine (DAB) was used for visualization, and hematoxylin was used for counterstaining. Microscopic analysis was conducted following dehydration, xylene transparency, and sealing.

### 2.7 Assessment of Mitochondrial Function

The ATP content and complex I/II/IV activities were detected using ATP Detection Kit (Beyotime, China) and Complex I, II, and IV activity assay kits (Solarbio, China), respectively. All steps were performed according to the manufacturer's instructions.

### 2.8 Dihydroethidium (DHE) Staining

Tissues were embedded in OCT embedding agent and cut into 10‐μm slices. Sections were then incubated with DHE (D807594, MACKLIN) for 30 min in the dark. After washing 3 times with phosphate‐buffered saline (PBS), the sections were mounted and observed under a microscope. For intracellular ROS detection, cells were treated with 4 μM DHE for 30 min in the dark. After washing with PBS, cells were observed under a microscope.

### 2.9 Primary Mouse Hippocampal Neuron Culture and Treatments

Primary hippocampal neurons were isolated from 12‐h newborn mice using an established protocol. Briefly, hippocampal tissues were digested with 1% papain for 30 min at 37°C and incubated with Dulbecco's modified Eagle's medium (DMEM) containing 10% fetal bovine serum (FBS) to terminate digestion. Isolated neurons were seeded in plates coated with poly‐D‐lysine and cultured at 37°C in Neurobasa medium (21103049, Gibico) containing 0.5 mM L‐glutamine and 2% B‐27. Neurons were transduced with lentivirus (SATB1^oe^ or SATB1^sh1/2^) for 72 h and incubated with hAmylin oligomers (10 μM) for the indicated time.

#### 2.9.1 Cell Counting Kit‐8 (CCK‐8) Assay

To measure cell viability, cells were treated with 10 μL of CCK‐8 solution (Biosharp, China) for 2 h at a 37°C incubator with 5% CO_2_. OD_450_ was detected using a microplate reader.

### 2.10 JC‐1 Staining

For JC‐1 staining, cells were incubated with 1 mL of JC‐1 working solution (BL711A, Biosharp) for 20 min at 37°C. Cells were washed twice with 1× JC‐1 staining buffer prior to microscopic analysis.

### 2.11 Real‐Time PCR

RNA was extracted using TRIpure reagent, and RNA concentration was determined by an ultraviolet spectrophotometer. Reverse transcription of RNA was next performed using All‐in‐One First strand super mix and dsDNase. Pangaea 3 real‐time fluorescence quantitative PCR instrument (Aperbio, China) was used for quantitative real‐time PCR analysis. The PCR mixture was made up of 1 μL cDNA, 0.5 μL SYBR GREEN, 10 μL Fast Taq plus PCR Master Mix, 0.5 μL forward primer, 0.5 μL reverse primer, and 7.5 μL ddH_2_O. Data analysis was conducted by the 2^–ΔΔ *C*^_t_ method. Primers used in this study are listed below (5′‐3″):

Mus SATB1 F: GCCAAAGGGCTCATCCA.

Mus SATB1 R: TGCGACCATTGTTCAGG.

Mus AKT3 F: GAAGAGTGGACGGAAGC.

Mus AKT3 R: ATGATGGGTTGTAGACG.

Mus β‐actin F: CATCCGTAAAGACCTCTATGCC.

Mus β‐actin R: ATGGAGCCACCGATCCACA.

### 2.12 Western Blot

Proteins were extracted using lysis buffer (Beyotime, China) and separated by sodium dodecyl sulfate–polyacrylamide gel electrophoresis (SDS‐PAGE) using 8% or 12% separation gel. The proteins were transferred onto a PVDF membrane and blocked for 1 h at room temperature. The membrane was incubated with primary antibodies at 4°C overnight and immersed in the secondary antibodies for 60 min at room temperature. Finally, protein bands were developed by ECL solution (P0018, Beyotime) and analyzed by Gel‐Pro‐Analyzer software. Antibodies used in this study were listed below: Anti‐SATB1 antibody (1:1000), anti‐AKT3 antibody (1:1000, 21641‐1‐AP, Proteintech, China), anti‐β‐actin (1:1000, sc‐47,778, Santa Cruz, China), goat anti‐rabbit IgG‐HRP (1:5000, Beyotime, A0208, China), and goat anti‐mouse IgG‐HRP (1:5000, Beyotime, A0216, China).

### 2.13 Caspase 3 Activity Detection

Commercial Caspase 3 activity assay kit (Solarbio Science & Technology Co Ltd., Beijing, China) was used to determine cellular caspase 3 activity using the established protocol.

#### 2.13.1 mRNA Sequencing (mRNA‐Seq)

After the evaluation of cognitive function, mouse hippocampal tissues were collected for mRNA‐seq, which was completed by Novogene (Beijing, China). To investigate the downstream gene of SATB1, hippocampal neurons expressing SATB1 or an empty vector were prepared and assigned for sequencing in Novogene (Beijing, China).

#### 2.13.2 Dual‐Luciferase Assay

HEK293T cells were co‐transfected with pGL3‐mus AKT3 promoter plasmid, mus SATB1 overexpressing (oe) plasmid, and Renilla plasmid. Luciferase activity was measured at 48 h post‐transfection, using a commercial detection assay kit (KGE3302, KeyGEN Biotechnology, Jiangsu, China).

#### 2.13.3 Chromatin Immunoprecipitation (ChIP) Assays

A ChIP assay was conducted using the ChIP Assay Kit (P2078) from Beyotime Biotech Inc. (Shanghai, China). In brief, chromatin complexes were incubated with anti‐SATB1 or IgG for immunoprecipitation. Subsequently, the immunoprecipitated DNA was subjected to reverse‐transcriptase PCR (RT‐PCR) analysis.

### 2.14 Statistical Analysis

Statistical analysis was performed using Graphpad Prism software. Normality of the distribution was examined by the Shapiro–Wilk test. Data with normal/Gaussian distribution were presented as mean ± SD. Comparison between the two groups was conducted by an unpaired Student's *t*‐test. One‐way ANOVA followed by Tukey's post‐test was used to compare three or more groups. Non‐normal data used corresponding non‐parametric tests. *p*‐Values less than 0.05 were considered significant.

## 3 Results

### 3.1 BHI of hAmylin Oligomers Impairs Cognitive Ability in Mice

To explore the effect of hAmylin oligomers on cognitive ability, Y maze and Morris Water maze tests were performed in C57BL/6 mice, 4 weeks after the BHI of hAmylin oligomers (Figure S1A). As shown in Figure S1B, the spontaneous alteration rate in hAmylin‐induced mice was remarkably decreased, suggesting impaired short‐term working memory in mice with hAmylin induction. Besides, hAmylin oligomers remarkably reduced their exploration time in the new arm, but increased the time spent in the starting arm and third arm (Figure S1C,D). Total distance and novel arm exploration frequency were decreased in mice upon hAmylin treatment (Figure S1E), suggesting damage to spatial reference memory. Morris Water maze tests were further carried out with a 5‐day navigation training test and a one‐day spatial exploration test (Figure S1F). As indicated in Figure S1G, the escape latency and swimming distance in hAmylin‐induced mice were remarkably increased when compared to those in the control group. On the contrary, the platform crossing number and target quadrant residence time were decreased in hAmylin‐induced mice (Figure S1H). All the results indicated the disorder of cognitive ability in hAmylin‐treated mice.

### 3.2 BHI of hAmylin Oligomers Causes Neuronal Damage in Mouse Hippocampus

Histopathological analyses were performed to detect neuronal damage in the hippocampus after hAmylin oligomer injection. As indicated in (Fig. S2A) number of TUNEL^+^NeuN^+^ cells in the hippocampus, especially in the DG area, was evidently increased in hAmylin‐induced mice. Similar results were obtained in Nissl staining (Fig. S2B). In addition, the degenerative neurons in the DG area, indicated by the FJB‐positive cells, were also augmented after hAmylin injection (Fig. S2C).

### 3.3 Effect of hAmylin Oligomer Injection on mRNA Expression Profile in Mouse Hippocampus Tissue

**FIGURE 1:**
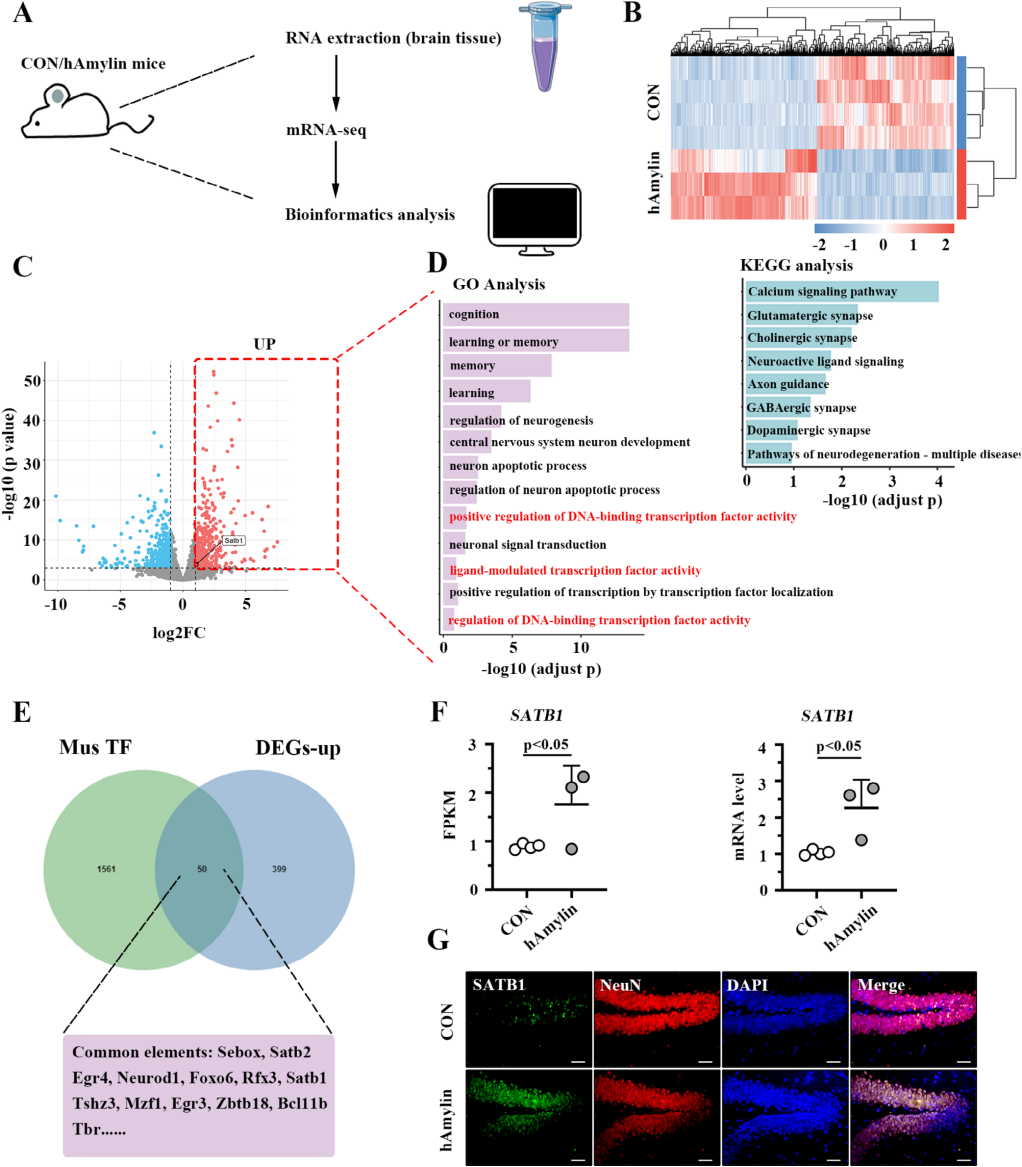
Effect of hAmylin oligomer injection on the mRNA expression profile in mouse hippocampus tissue. A. Representation of the mRNA‐seq workflow. B. Heatmap showing differentially expressed genes. C. Volcano plot presenting differentially expressed genes. D. Left: GO terms enriched by the upregulated genes. Right: KEGG pathways enriched by the upregulated genes. E. Venn diagram indicating overlaps between Mus TF (AnimalTFBD) and upregulated genes. F. Relative mRNA level of SATB1 in the mouse hippocampus. G. Representative immunofluorescence images of SATB1/NeuN‐stained sections from the mouse hippocampal DG area. Bar: 50 μm. GO: Gene Ontology; KEGG: Kyoto Encyclopedia of Genes and Genomes; TF: Transcription factor; DG: Dentate Gyrus.

Hippocampal tissues from control mice and hAmylin mice were subjected to mRNA sequencing (Figure 1A), and differentially expressed genes (DEGs) were identified according to the standard of |Log_2_FC| > 1 and *p* < 0.001. Figure 1B presented a clustering heat map of all DEGs, and a total of 421 downregulated genes and 449 upregulated genes were determined (Figure 1C). GO and KEGG enrichment analyses (Figure 1D) indicated that these upregulated genes displayed enrichment in the GO term “positive regulation of DNA‐binding transcription factor activity,” “ligand‐modulated transcription factor activity,” “cognition,” “learning or memory,” “memory,” and “learning.” Thus, Mus transcription factors (TF) were downloaded from AnimalTFBD, and 50 overlaps (including SATB1) were identified among Mus TF and upregulated DEGs (Figure 1E). Meanwhile, the overexpression of SATB1 in hAmylin‐induced hippocampus was determined by the fragments per kilobase of exon model per million mapped fragments (FPKM) data derived from sequencing (Figure 1F, left) and results of real‐time PCR analysis (Figure 1F, right), using tissue samples with the same source as sequencing samples. Immunofluorescence staining assay (Figure 1G) on the DG area of the mouse hippocampus further indicated the localization of SATB1 in neurons, highlighting the critical role of SATB1 in regulating neuronal functions.

### 3.4 SATB1 Knockdown Aggravates hAmylin Oligomer‐Induced Cognitive Impairments

**FIGURE 2:**
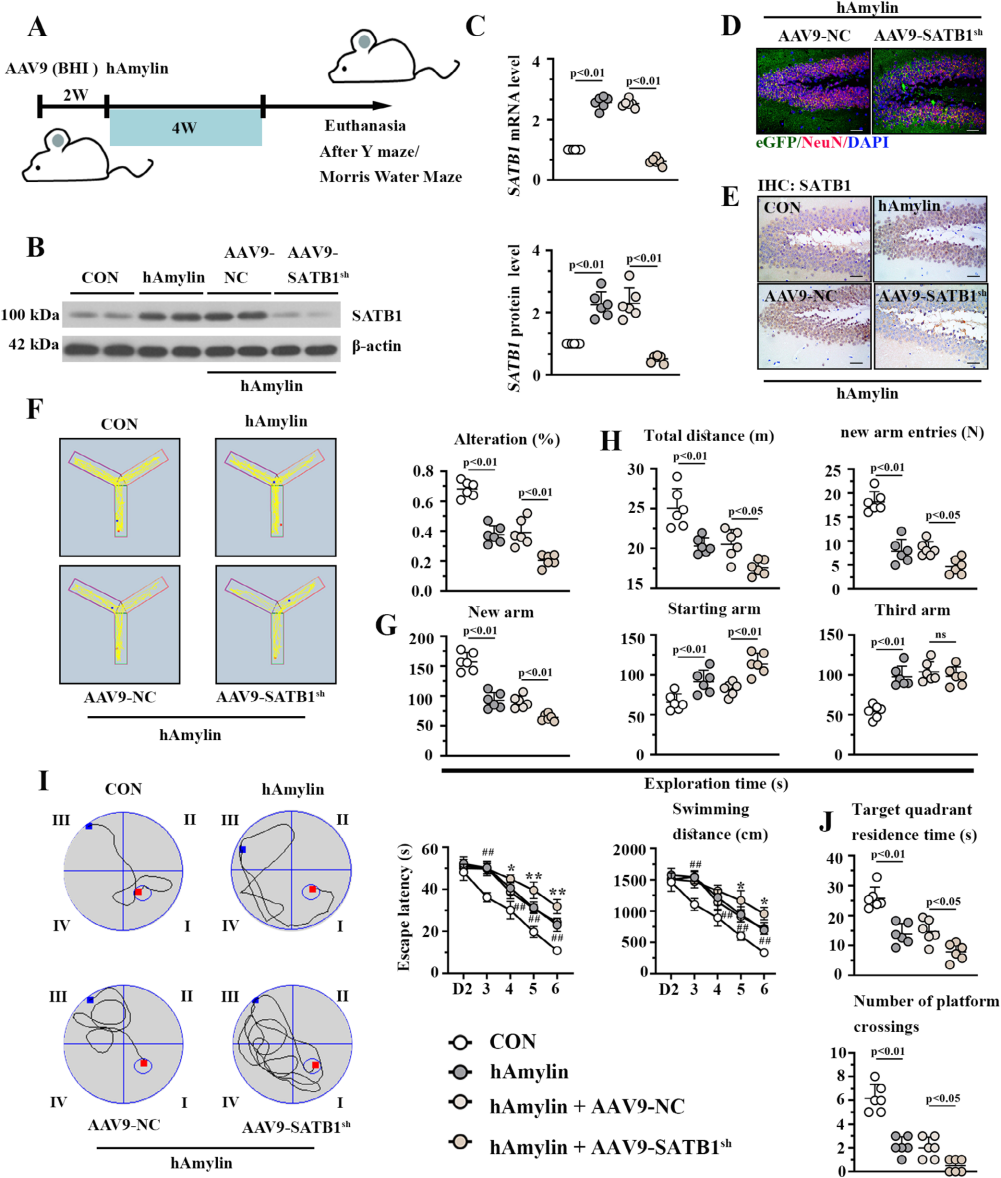
SATB1 Knockdown aggravates hAmylin oligomer‐induced cognitive impairments. A. Brief presentation about the workflow of in vivo experiments. B. Representative immunoblotting images of SATB1. C. Relative mRNA level of SATB1. D. GFP and NeuN immunofluorescence images of the mouse hippocampal DG region. Bar: 50 μm. E. Immunohistochemical images of the mouse hippocampal DG region using the SATB1 antibody. Bar: 50 μm. F. Spontaneous alteration percentage in the Y‐maze test, along with a representative movement trajectory map of the mice on the left. G. Exploration time of mice in different arms. H. Total distance and new arm entries in the Y‐maze test. I. Left: Representative trace plot on the last day of navigation test. Right: The escape latency and swimming distance in the navigation Test. J. Target quadrant residence time and platform crossing numbers in Spatial Exploration Test. GFP: Enhanced green fluorescent protein; ns: No significance; NC: Negative control shRNA; SATB1^sh^: SATB1 shRNA; DG: Dentate Gyrus.

Two weeks before hAmylin injection, the mice received BHI of AAV9 particles carrying SATB1 shRNA and eGFP (Figure 2A). The protein and mRNA levels of SATB1, determined by western blotting and real‐time PCR, were significantly downregulated in the hippocampus after AAV9‐SATB1^sh^ infection (Figure 2B,C). Besides, immunofluorescence images in Figure 2D displayed eGFP‐positive staining in the neurons, validating the infectivity of AAV9 in neurons of the hippocampal DG area. Immunohistochemistry assay further determined the downregulation of SATB1 in the DG area of mice after AAV9‐SATB1^sh^ infection (Figure 2E). In the Y maze test, mice with SATB1 knockdown exhibited reduced spontaneous alternation (Figure 2F–H), decreased exploration time in the new arm, diminished total distance, and reduced new arm entries. In the Morris Water maze test, the escape latency and swimming distance were obviously increased in mice with SATB1 knockdown (Figure 2I). Additionally, SATB1 knockdown reduced the platform crossing number and target quadrant residence time in hAmylin‐treated mice (Figure 2J). In summary, SATB1 knockdown induced further cognition decline in hAmylin‐induced mice.

### 3.5 SATB1 Knockdown Aggravates hAmylin Oligomer‐Induced Neuronal Damage

**FIGURE 3:**
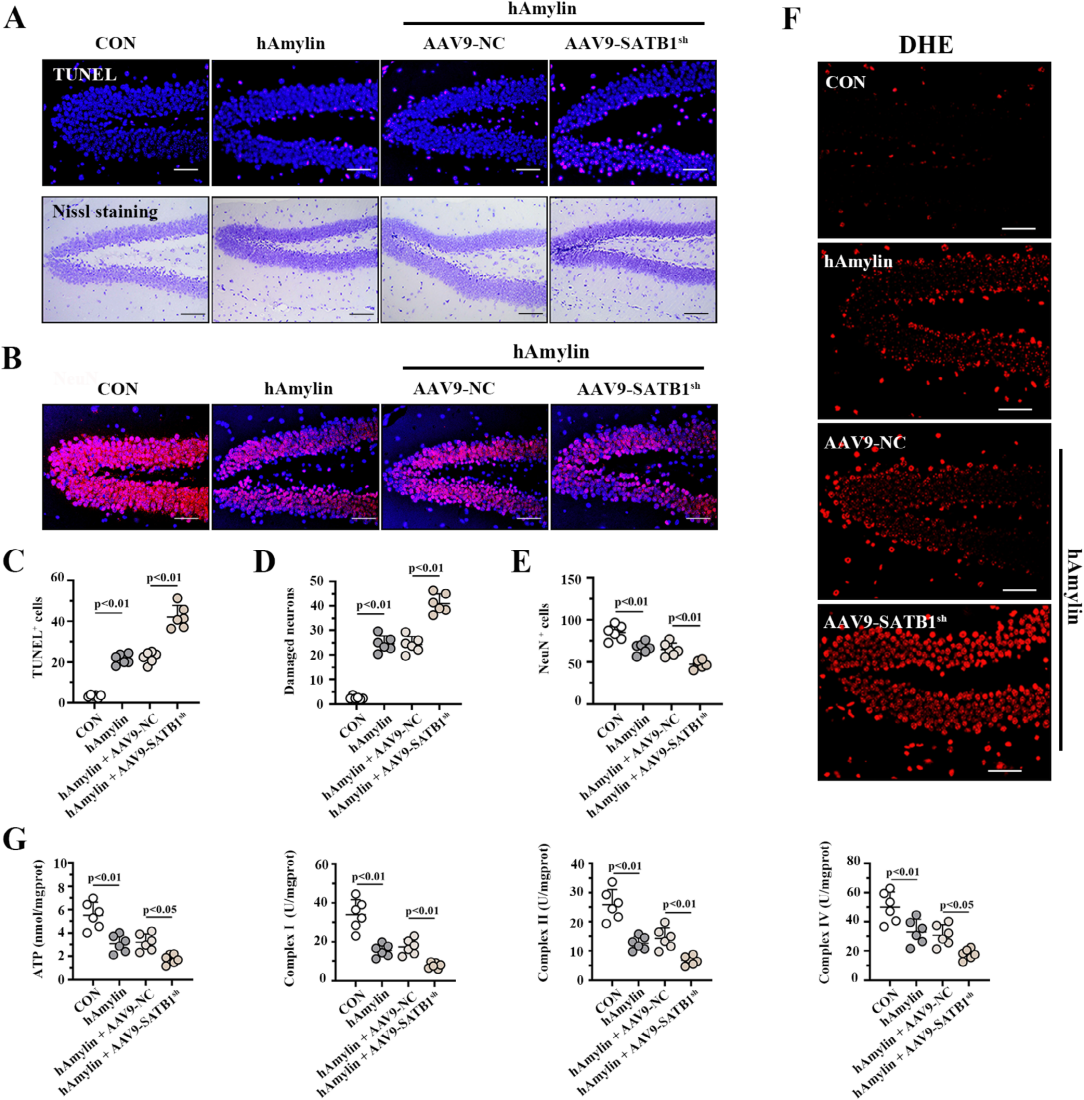
SATB1 knockdown aggravates hAmylin‐induced neuronal damage. A. Results of TUNEL/Nissl staining on the section of mouse hippocampus DG area; bar = 50 μm/100 μm. B. NeuN immunofluorescence images of mouse hippocampus DG area; bar = 50 μm. C‐E. Quantitative analysis of TUNEL^+^ cells, damaged neurons, and NeuN^+^ cells in the mouse hippocampus DG area. F. Images of DHE staining on the DG area of the mouse hippocampus; bar = 50 μm. G. ATP content and complex I/II/IV activities in mouse hippocampus. NC: Negative control shRNA; SATB1^sh^: SATB1 shRNA; DG: Dentate Gyrus.

Histopathological analyses were performed to measure neuronal damage in hAmylin oligomer‐induced hippocampus after SATB1 knockdown. As shown in Figure 3A, TUNEL‐positive cells and damaged neurons were obviously increased in the DG area of hAmylin mice after SATB1 knockdown. Besides, NeuN‐positive cells were decreased in the DG area of mice upon SATB1 knockdown (Figure 3B), validating the neurotoxicity of hAmylin oligomers. The corresponding quantitative analyses to Figure 3A,B are presented below (Figure 3C–E), and it is further determined that hAmylin oligomers elevated ROS levels (Figure 3F), reduced ATP levels, and inhibited complex I/II/IV activities (Figure 3G) in the hippocampus of mice. However, SATB1 knockdown further aggravated it.

### 3.6 SATB1 Inhibits Apoptosis Induced by hAmylin Oligomers in Primary Hippocampal Neurons

**FIGURE 4:**
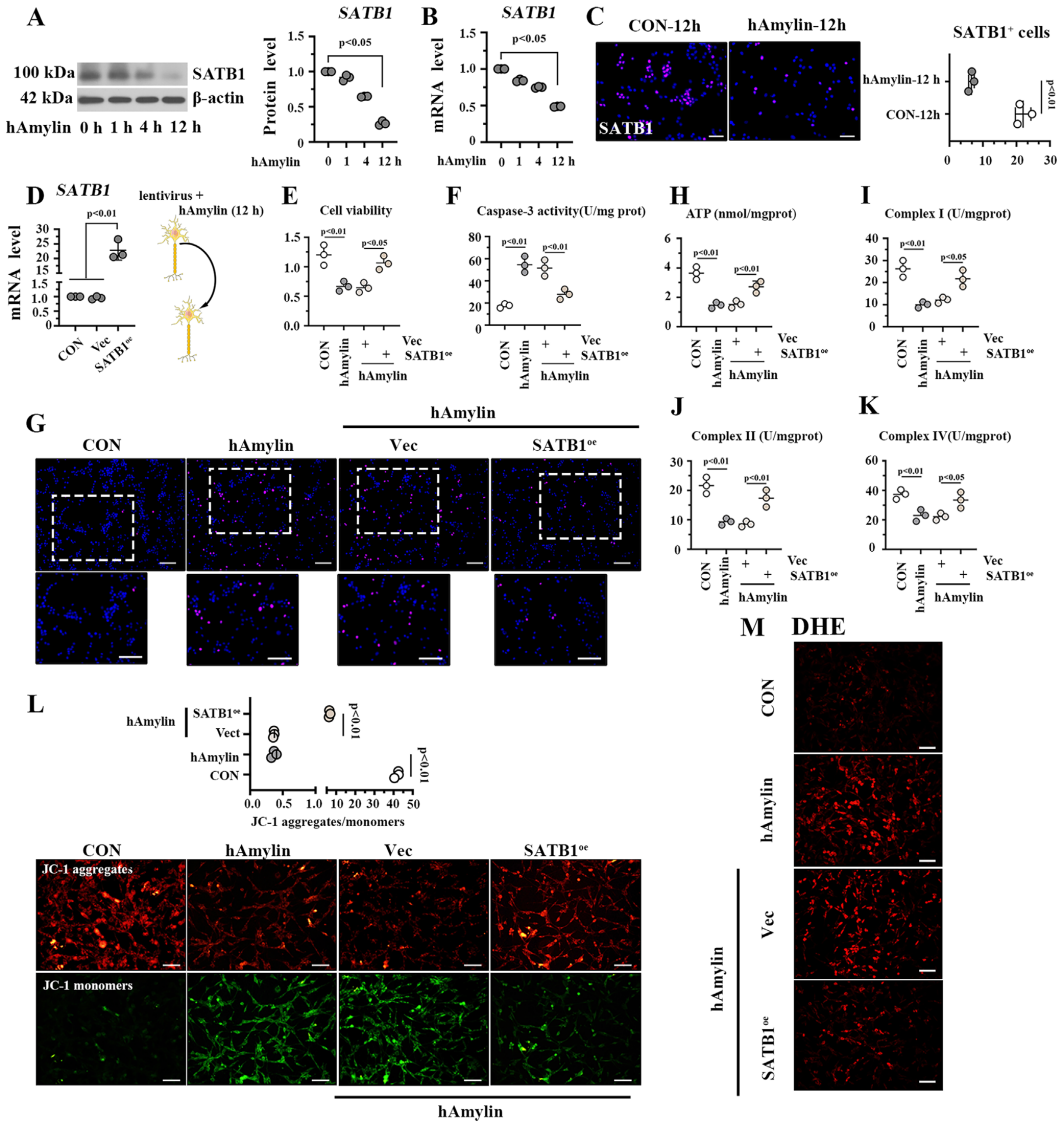
SATB1 inhibits apoptosis and mitochondrial dysfunction induced by hAmylin oligomers in primary hippocampal neurons. A‐B. Relative mRNA and protein levels of SATB1 in primary mouse hippocampal neurons treated with 10 μM hAmylin for 0, 1, 4, and 12 h. C. Immunofluorescence images of SATB1 in primary hippocampal neurons treated with 10 μM hAmylin for 12 h; bar = 50 μm. D. Relative mRNA level of SATB1 in primary hippocampal neurons overexpressing SATB1. E. Workflow of the in vitro assay and cell viability of primary neurons with SATB1 overexpression and hAmylin treatment. F. Caspase 3 activity in neurons with SATB1 overexpression and hAmylin treatment. G. TUNEL staining results of primary neurons with SATB1 overexpression and hAmylin treatment; bar = 100 μm. H‐K. ATP content and complex I/II/IV activities in primary mouse neurons with SATB1 overexpression and hAmylin treatment. L. JC‐1 staining assay for detecting mitochondrial membrane potential in hAmylin‐induced neurons with SATB1 overexpression; bar = 100 μm. M. ROS levels in primary hippocampal neurons, which were determined by DHE staining; bar = 100 μm. Vec: Empty vector; SATB1^oe^: SATB1 overexpression.

Primary hippocampal neurons were identified by cellular morphology (Fig. S3) and incubated with hAmylin oligomers for 0, 1, 4, and 12 h. The expression levels of SATB1 in primary neurons were significantly reduced upon hAmylin induction (Figure 4A–C). Then, SATB1 was overexpressed or knocked down in primary neurons using lentivirus infection (Figure 4D, Fig. S4A). To explore the effect of SATB1 on hAmylin‐induced neurons, the infected neurons were exposed to hAmylin oligomers. As shown in Figure 4E,F. SATB1 overexpression improved cell viability and decreased caspase 3 activity in hAmylin‐induced neurons. SATB1 restored hAmylin‐induced neuronal apoptosis, with decreased TUNEL^+^ cells (Figure 4G). On the contrary, SATB1 silencing reduced cell viability in hAmyin‐induced neurons (Fig. S4B) and exacerbated hAmyin‐induced cell apoptosis, with elevated caspase 3 activity (Fig. S4C) and augmented TUNEL^+^ cells (Fig. S4D).

### 3.7 SATB1 Alleviates hAmylin Oligomer‐Induced Mitochondrial Dysfunction in Primary Hippocampal Neurons

As presented in Figure 4H–K, hAmylin oligomers reduced ATP level and inhibited complex I/II/IV activity in primary hippocampal neurons (Figure 4I), while SATB1 overexpression restored these changes. Moreover, neurons overexpressing SATB1 exhibited decreased JC‐1 aggregates and increased JC‐1 monomers, which suggests that SATB1 elevated mitochondrial membrane potential in hAmylin‐induced neurons (Figure 4L). The ROS levels, determined by DHE staining, were obviously elevated upon hAmylin treatment and reduced after SATB1 overexpression (Figure 4M). More inspiringly, SATB1 knockdown further aggravated mitochondrial dysfunctions in hAmylin‐induced neurons, with reduced mitochondrial membrane potential (Fig. S4E), augmented ROS level (Fig. S4F), decreased ATP level, and attenuated complex I/I/IV activities (Fig. S4G). Collectively, SATB1 displays a protective effect on mitochondrial dysfunction in hAmylin‐induced neurons.

### 3.8 AKT3: Potential Downstream Target of SATB1

**FIGURE 5:**
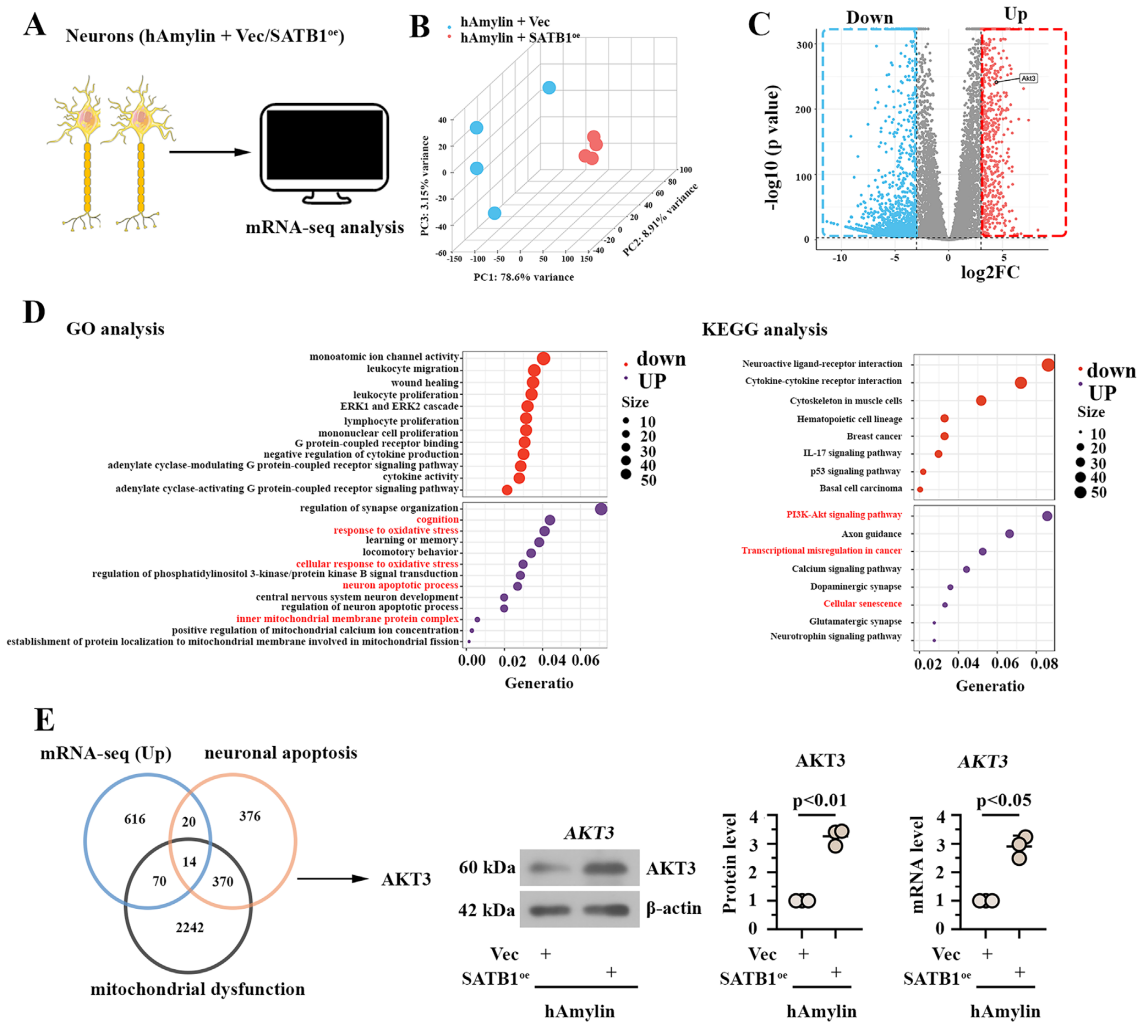
AKT3: Potential downstream target of SATB1. A. Neurons with hAmylin treatment and SATB1 overexpression were subjected to mRNA sequencing to identify the potential downstream target of SATB1. B. Principal component analysis of all cell samples. C. Differentially expressed genes presented by a volcano map and identified by the threshold of |Log_2_FC| > 3 and *p*‐value < 0.001. D. GO terms and KEGG pathways enriched by differentially expressed genes. E. Schematic diagram depicting the screening process of AKT3. The AKT3 level in neurons overexpressing SATB1 was measured by real‐time PCR and western blotting. ns: No significance; Vec: Empty vector; SATB1^oe^: SATB1 overexpression; GO: Gene Ontology; KEGG: Kyoto Encyclopedia of Genes and Genomes.

To investigate the potential downstream target of SATB1, mRNA sequencing was performed on hAmylin‐neurons expressing SATB1 or vector (Figure 5A). Significant intergroup differences were identified among the two groups (Figure 5B), and the DEGs were presented as a Volcano plot with the threshold of |Log2FC| > 3 and *p* < 0.001(Figure 5C). We subjected the DEGs to GO and KEGG analyses, and upregulated DEGs were enriched in the GO term “cognition” and “oxidative stress” More importantly, they also exhibited involvement in “PI3K‐AKT signaling pathway”. SATB1 has been documented as a transcriptional activator [20]. Therefore, an intersection between the upregulated DEGs, “neuronal apoptosis protein encoding genes” and “mitochondrial dysfunction protein encoding genes” was performed. As shown in Figure 5E, 14 common genes, including AKT3, were identified in the intersection. Using real‐time PCR and western blotting analysis, AKT3 expression was determined to be significantly upregulated in hAmylin‐induced neurons after SATB1 overexpression.

### 3.9 SATB1 Regulates Neuronal Apoptosis and Mitochondrial Function Through AKT3

**FIGURE 6:**
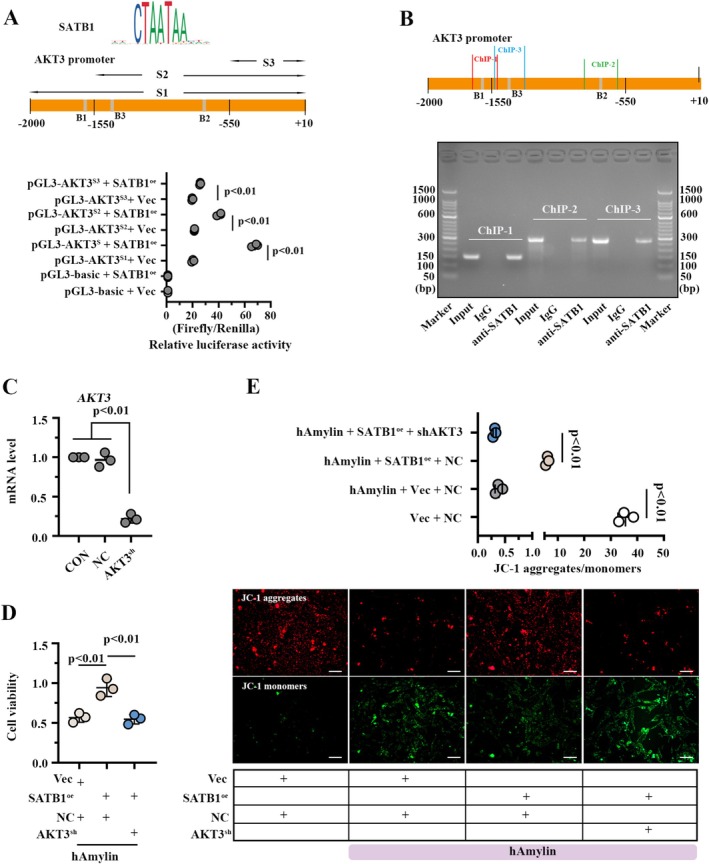
SATB1 regulates neuronal damage and mitochondrial function through AKT3. A. SATB1 binding sites in AKT3 promoter were predicted by Jaspar database (https://jaspar.elixir.no/). Dual‐luciferase assay was performed to verify the binding between SATB1 and AKT3 promoter. B. ChIP‐PCR was conducted to validate the binding of SATB1 with the AKT3 promoter. C. AKT3 mRNA level in the neurons with AKT3 knockdown. D. Cell viability of neurons with hAmylin induction and AKT3 knockdown. E. JC‐1 staining analysis for detecting mitochondrial membrane potential in the neurons; bar = 100 μm. ns: No significance; Vec: Empty vector; SATB1^oe^: SATB1 overexpression; NC: Negative control shRNA; AKT3^sh^: AKT3 shRNA.

Dual‐luciferase assay and ChIP assay verified the binding between SATB1 and AKT3 promoter (Figure 6A,B). To further determine whether AKT3 accounts for SATB1‐mediated protection against hAmylin‐induced mitochondrial dysfunctions and neuronal damage, AKT3 was knocked down or overexpressed in neurons using lentivirus (Figure 6C, Fig. S4H). SATB1 increased cell viability (Figure 6D) and elevated mitochondrial membrane potential (Figure 6E) in hAmylin‐induced neurons, while these effects were reversed by AKT3 knockdown. Additionally, AKT3 overexpression promoted cell viability (Fig. S4I) and elevated mitochondrial membrane potential (Fig. S4J) in hAmylin neurons with SATB1 knockdown. In summary, AKT3 mediated the function of SATB1 in regulating mitochondrial function and cell viability.

## 4 Discussion

Amylin has drawn extensive attention in diabetes and AD, while the effect of hAmylin oligomers on cognitive function in normal mice has not been fully explored. As reported, the hippocampus is a core region responsible for cognitive function, and the deposition of hAmylin has been observed in the hippocampus of high‐fat diet (HFD)‐induced hIAPP transgenic mice [9]. Thus, hAmylin oligomers were bilaterally injected into the hippocampus of mice, and this study determined that heterologous amylin induced cognitive decline in mice following the bilateral hippocampal injections. Besides, the DG area in the hippocampus was found to be severely damaged after hAmylin injection, according to the results of histological examination. In line with this finding, a previous study has reported that hAmylin injection into the right lateral ventricle induces neuronal apoptotic cell deaths in the hippocampal DG region [12]. The DG area is a crucial part of hippocampal structures and has profound effects on learning, memory, and neurogenesis [30, 31]. Although there are structural differences between human DG and mouse DG, the two are highly conserved in the core mechanisms, such as memory distinguishing and spatial navigation. Additionally, impaired neurogenesis in the DG area has been widely observed in the development of neurodegenerative diseases. Accordingly, neurons in the DG area are significantly decreased among AD patients, thereby resulting in memory impairments [32, 33]. Thus, the pathological change in the DG area of hAmylin mice was in the spotlight, and our findings indicated that hAmylin oligomers induced neuronal degeneration, neuronal apoptosis, and mitochondrial dysfunction in the DG area. Consistently, human amylin activates the caspase cascade to evoke apoptosis of beta cells [34]. Fibrillogenic human amylin drives apoptotic cell death in human mesangial cells [35]. Human amylin shares a toxicity pathway with myloid beta (Aβ) via inducing mitochondrial dysfunction [36]. hAmylin deposition in the brain exacerbates neuronal damage and mitochondrial dysfunction in AD development [11]. Besides, most researchers believe that Amylin's neurotoxicity is attributed to its oligomers rather than monomers [37], further supporting our current findings.

Following the injection of hAmylin oligomers, SATB1 is upregulated in the hippocampus of mice, while the expression level of SATB1 was significantly reduced in primary hippocampal neurons after hAmylin stimulation. SATB1 knockdown promotes mitochondrial dysfunction in T cells via inducing mitochondrial ROS [38]. SATB1 regulates the expression of mitochondrial fusion protein MFN2 to preserve mitochondrial morphological integrity and cell survival [39]. SATB1 reduced ATP production and inhibited mitochondrial enzymatic activity (complexes I) in CD4 T cells [19]. SATB1 restrains tumor apoptotic cell deaths by inducing anti‐apoptotic proteins [40]. In line with the above research, AAV9‐mediated SATB1 knockdown aggravated neuronal damages and cognitive impairment in hAmylin‐induced mice, with increased apoptotic neuronal deaths, severe mitochondrial dysfunction, and serious cognition decline. SATB1 overexpression protected against hAmylin‐induced cell apoptosis and mitochondrial dysfunction in hippocampal neurons, while SATB1 knockdown aggravated it. All the findings suggest the protective role of SATB1 in hAmylin oligomer‐induced neuronal damage and cognition decline. As for the contradiction to SATB1 expression change in hAmylin‐induced mouse hippocampus and primary hippocampal neurons, it is believed to be attributed to the complexity of the in vivo microenvironment, and this response is operative in vivo, buffers against acute perturbations, whereas isolated cells in a simplified environment can't mount such a response. Additionally, similar phenomena have been observed in the development of sepsis‐induced cardiomyopathy [41] and AD [42], coordinating with our findings.

AKT3, the potential target gene of SATB1, was identified through mRNA sequencing. As a member of the AKT family, AKT3 has displayed protective effects on a range of neuropathic diseases. Briefly, the spatial cognitive ability in AKT3‐deficient mice was extremely impaired [22], while AKT3 overexpression exerts a beneficial effect on the survival of spinal motor neurons in a mouse model of familial amyotrophic lateral sclerosis [23]. In this study, the binding between SATB1 and the AKT3 promoter was verified using a dual‐luciferase activity assay and the ChIP method. Besides, SATB1 induced AKT3 transcription via this binding. It is worth mentioning that there are potential synergies between SATB1 and AKT3 in terms of regulating cell apoptosis and mitochondrial function. AKT3 plays an indispensable role in preserving mitochondrial function [24, 43]. Targeting AKT3 protects cochlea hair cells from radiation‐induced apoptosis [44]. AKT3 acts as a major downstream effector for neuroprotection against motoneuronal cell death [45]. AKT3 inhibits the apoptosis of chondrocytes during osteoarthritis development [46]. Herein, this study demonstrates that SATB1 protects hippocampal neurons from hAmylin‐induced mitochondrial dysfunction and apoptosis through AKT3, suggesting that there is a regulatory axis among hAmylin, SATB1, and AKT3.

Nonetheless, the findings of this study have to be seen in light of some limitations. Although our findings identify AKT3 as a critical downstream effector of SATB1, we acknowledge that there might be other downstream targets. Besides, only male mice were used in this study due to the effect of estrogen changes on cognitive performance. Although this method enhances the reproducibility of our experimental results and avoids the fluctuations caused by hormone changes, the role of SATB1 in hAmylin‐induced female mice remains fully explored, and it will be our next topic in the future.

In conclusion, this research verifies that bilateral hippocampal injections of hAmylin oligomers induce cognitive dysfunction and neuronal damage in mice. SATB1 resists hAmylin‐induced neuronal damage through the transcriptional regulation of AKT3.

## Author Contributions

N.Z. and Y.X. contributed to the conception of this work, Y.X. and W.Z. performed the experiments, Y.X., Z.Y., and X.Q. analyzed and interpreted the data, and W.Z., Z.Y., and T.Z. drafted the manuscript. All authors reviewed and edited the manuscript.

## Funding

This study was funded by the Key Research and Development Program of Hebei Province (23377708D), Hebei Province Government‐funded Excellent Talents Project in Clinical Medicine (ZF2023029), Science and Technology Research Project of Hebei Colleges (BJK2023072), and the Postdoctoral Science Foundation Project of the Chongqing Natural Science Foundation (CSTB2023NSCQ‐BHX0119).

## Consent

All animal experiments were performed with the approval of the Ethics Committee of the First Hospital of Hebei Medical University (S00668).

## Conflicts of Interest

The authors declare no conflicts of interest.

## Supporting information


**Figure S1:** Bilateral hippocampal injections (BHI) of hAmylin oligomers impair cognitive ability in mice. A. Workflow presentation. B. Y maze test was used to evaluate the short‐term working memory ability of mice. Left panel: Representative movement trajectory map of mice. Right panel: Quantification of spontaneous alteration percentage. C. Left panel: Representative trace plot of the spatial reference memory ability test. D. Right panel: Exploration time of mice in different arms. E. Total distance traveled by mice and number of new arm entries in the spatial reference memory ability test. F. Brief illustration about Morris Water Maze. G. Up: Representative trace plot of navigation test on D6. Down: The escape latency and swimming distance in the navigation test. H. Up: Representative trace plot of the spatial exploration test on D7. Down: Target quadrant residence time and platform crossing numbers in the spatial exploration test.
**Figure S2:** Bilateral hippocampal injections (BHI) of hAmylin oligomers cause neuronal damage in the mouse hippocampus. A. TUNEL and NeuN double immunofluorescence staining images of mouse hippocampus; bar = 500 μm (40×), 50 μm (400×). B. Nissl staining images of mouse hippocampus; bar = 500 μm (40×), 100 μm (200×). C. Fluoro Jade B staining images of hippocampus; bar = 500 μm (40×), 50 μm (400×).
**Figure S3:** Identification of mouse primary hippocampal neuron. Representative cellular morphology of primary hippocampal neurons was observed under a microscope (bar: 50 μm).
**Figure S4:** SATB1 knockdown promoted cell apoptosis and mitochondrial dysfunctions in hAmylin oligomer‐induced primary hippocampal neurons. A. SATB1 was downregulated in primary hippocampal neurons via lentivirus infection. B‐D. Following the infection and hAmylin oligomer stimulation, cell viability and apoptosis of hAmylin‐induced neurons were measured. E. Representative images of JC‐1‐stained neurons. Bar = 100 μm. Quantitative analysis of fluorescence intensity (aggregates/monomers) is presented below. F. Representative images of DHE‐stained neurons; bar = 100 μm. G. ATP content and complex I/II/IV activities in hAmylin‐induced neurons with SATB1 knockdown. H. AKT3 was overexpressed in primary hippocampal neurons via lentivirus infection. I. Cell viability of hAmylin‐induced neurons with SATB1 knockdown and AKT3 overexpression. J. Representative images of JC‐1‐stained neurons; bar = 100 μm. Quantitative analysis of fluorescence intensity (aggregates/monomers) is presented on the right.

## Data Availability

The data that support the findings of this study are available from the corresponding author upon reasonable request.
