# Supplementary material for: SATB1 Exhibits a Protective Role in HAmylin‐Oligomer‐Induced Neuronal Damage and Cognition Decline
Source: CNS Neurosci Ther. 2026 Feb 13;32(2):e70781. doi: 10.1002/cns.70781 (PMC12904839; doi:10.1002/cns.70781)
Supplement: Supplementary file 1 — Figure S1: Bilateral hippocampal injections (BHI) of hAmylin oligomers impair cognitive ability in mice. A. Workflow presentation. B. Y maze test was used to evaluate the short‐term working memory ability of mice. Left panel: Representative movement trajectory map of mice. Right panel: Quantification of spontaneous alteration percentage. C. Left panel: Representative trace plot of the spatial reference memory ability test. D. Right panel: Exploration time of mice in different arms. E. Total distance traveled by mice and number of new arm entries in the spatial reference memory ability test. F. Brief illustration about Morris Water Maze. G. Up: Representative trace plot of navigation test on D6. Down: The escape latency and swimming distance in the navigation test. H. Up: Representative trace plot of the spatial exploration test on D7. Down: Target quadrant residence time and platform crossing numbers in the spatial exploration test. Figure S2: Bilateral hippocampal injections (BHI) of hAmylin oligomers cause neuronal damage in the mouse hippocampus. A. TUNEL and NeuN double immunofluorescence staining images of mouse hippocampus; bar = 500 μm (40×), 50 μm (400×). B. Nissl staining images of mouse hippocampus; bar = 500 μm (40×), 100 μm (200×). C. Fluoro Jade B staining images of hippocampus; bar = 500 μm (40×), 50 μm (400×). Figure S3: Identification of mouse primary hippocampal neuron. Representative cellular morphology of primary hippocampal neurons was observed under a microscope (bar: 50 μm). Figure S4: SATB1 knockdown promoted cell apoptosis and mitochondrial dysfunctions in hAmylin oligomer‐induced primary hippocampal neurons. A. SATB1 was downregulated in primary hippocampal neurons via lentivirus infection. B‐D. Following the infection and hAmylin oligomer stimulation, cell viability and apoptosis of hAmylin‐induced neurons were measured. E. Representative images of JC‐1‐stained neurons. Bar = 100 μm. Quantitative analysis of fluorescence intensity (aggregat [file CNS-32-e70781-s001.docx]

**Supplementary Material for Review**

**
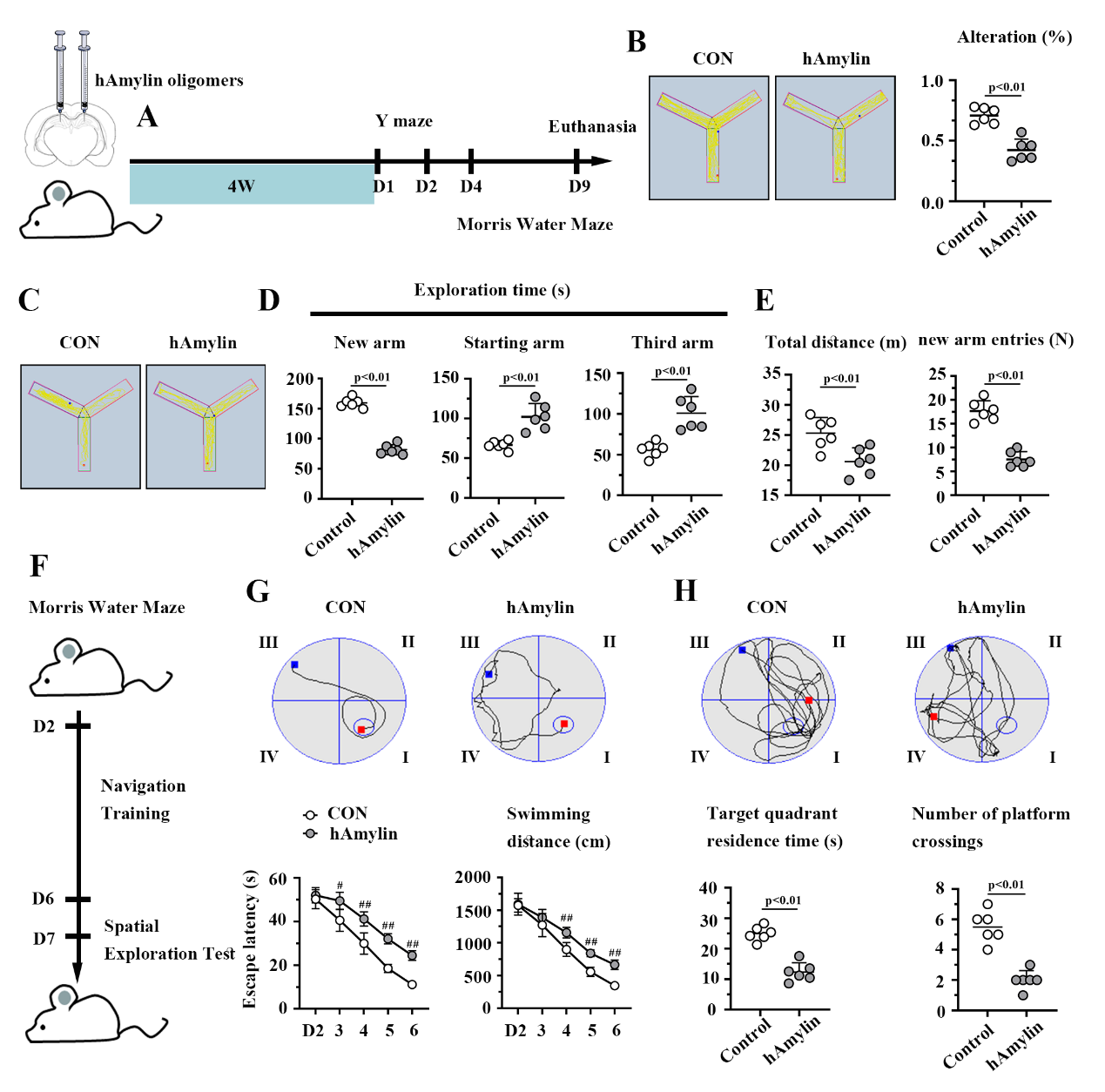
**

**Figure S1. Bilateral hippocampal injections (BHI) of hAmylin oligomers impairs cognitive ability in mice.** A. Work flow presentation. B. Y maze test was used to evaluate short-term working memory ability of mice. Left panel: Representative movement trajectory map of mice. Right panel: Quantification of spontaneous alteration percentage. C. Left panel: Representative trace plot of spatial reference memory ability test. D. Right panel: Exploration time of mice in different arms. E. Total distance traveled by mice and number of new arm entries in the spatial reference memory ability test. F. Brief illustration about Morris Water Maze. G. Up: Representative trace plot of navigation test on D6. Down: The escape latency and swimming distance in navigation test. H. Up: Representative trace plot of spatial Exploration Test on D7. Down: Target quadrant residence time and platform crossing numbers in Spatial Exploration Test.

**
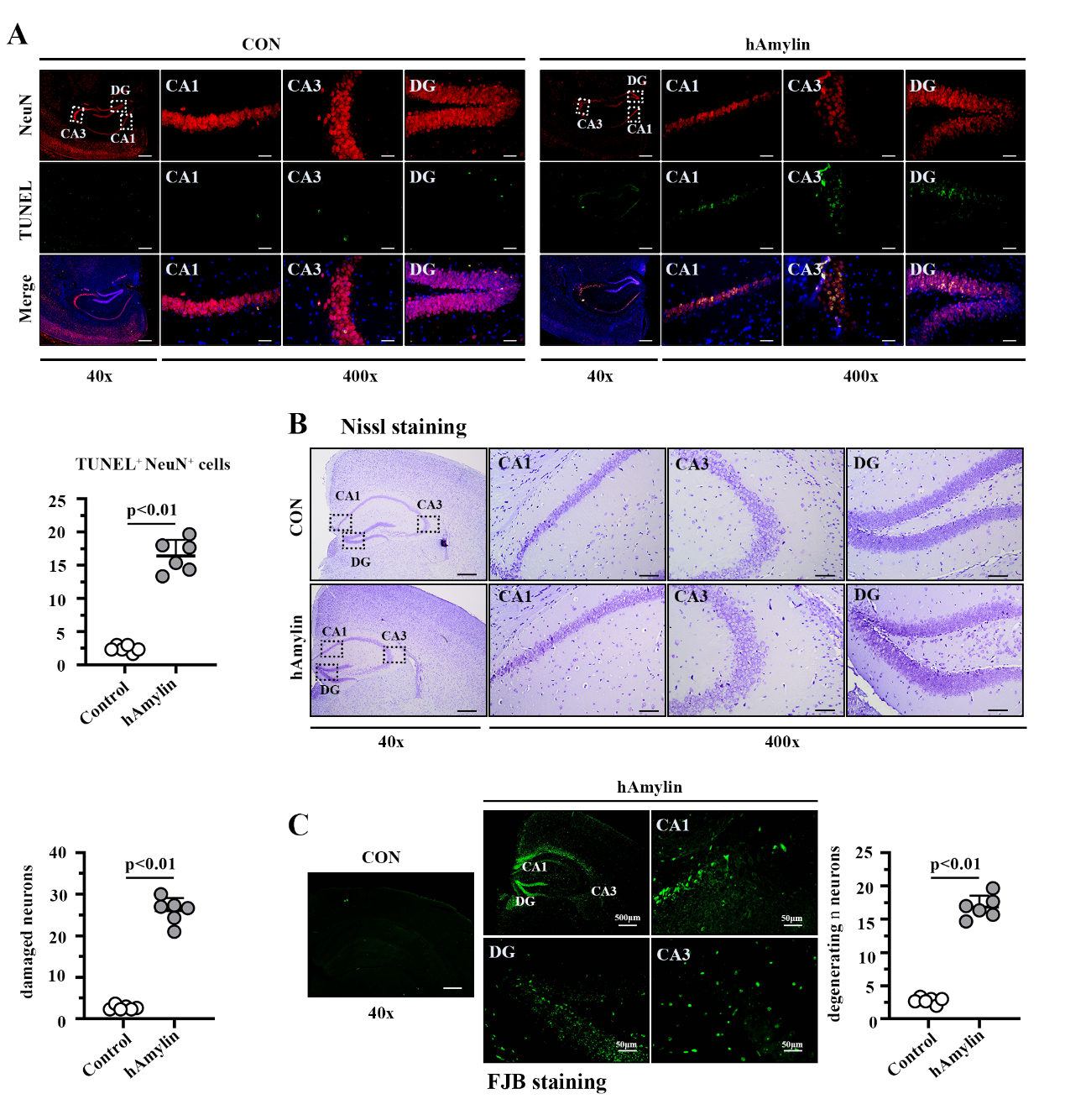
**

**Figure S2. Bilateral hippocampal injections (BHI) of hAmylin oligomers causes neuronal damage in mouse hippocampus**

A. TUNEL and NeuN double immunofluorescence staining images of mouse hippocampus, bar=500 μm (40×), 50 μm (400×). B. Nissl staining images of mouse hippocampus, bar=500 μm (40×), 100 μm (200×). C. Fluoro Jade B staining images of hippocampus, bar=500 μm (40×), 50 μm (400×).

**
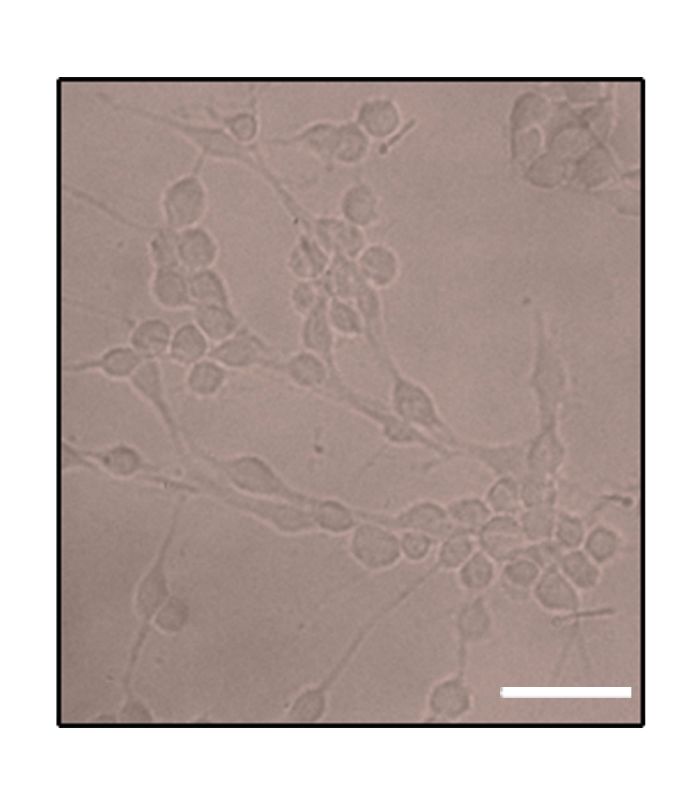
**

**Figure S3 Identification of mouse primary hippocampal neuron.** Representative cellular morphology of primary hippocampal neurons were observed under a microscope (Bar: 50 μm).


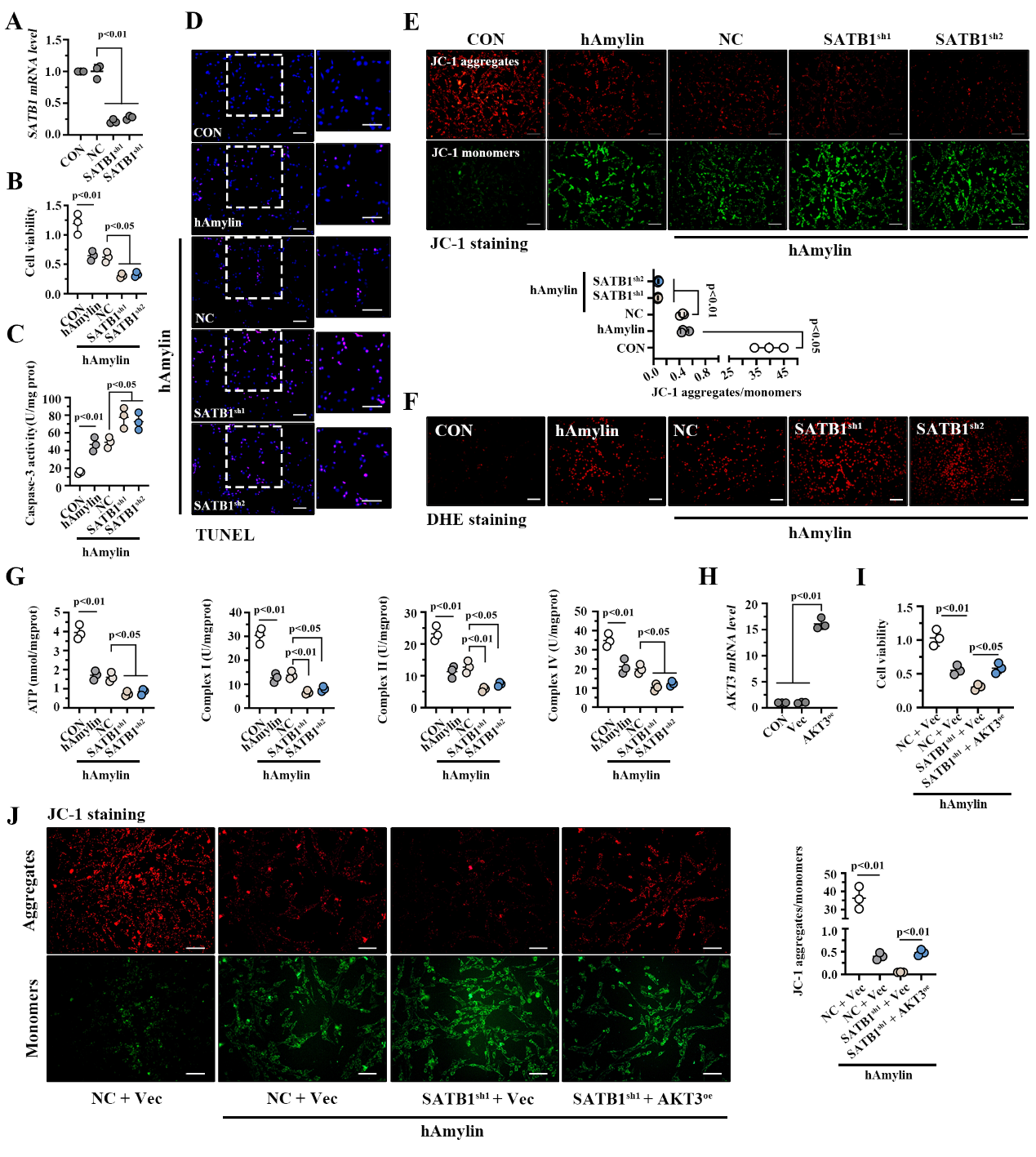


**Figure S4 SATB1 knockdown promoted cell apoptosis and mitochondrial dysfunctions in hAmylin oligomer-induced primary hippocampal neurons.** A. SATB1 was downregulated in primary hippocampal neurons via lentivirus infection. B-D. Following the infection and hAmylin oligomer stimulation, cell viability and apoptosis of hAmylin-induced neurons were measured. E. Representative images of JC-1 stained neurons. Bar= 100 μm. Quantitative analysis of fluorescence intensity (Aggregates/monomers) was presented below. F. Representative images of DHE-stained neurons. Bar= 100 μm. G. ATP content and Complex I/II/IV activity in hAmylin-induced neurons with SATB1 knockdown. H. AKT3 was overexpressed in primary hippocampal neurons via lentivirus infection. I. Cell viability of hAmylin-induced neurons with SATB1 knockdown and AKT3 overexpression. J. Representative images of JC-1 stained neurons. Bar= 100 μm. Quantitative analysis of fluorescence intensity (Aggregates/monomers) was presented on the right.
